# Assessing prevalence and risk factors for REM sleep behavior disorder among patients with inflammatory bowel disease

**DOI:** 10.1038/s41531-025-01051-7

**Published:** 2025-10-01

**Authors:** Vijaya L. Reddy, Zihan Chen, Sohail Dewain, Michelle Joo, Maya Bellomo, Erika Renkl, Sapna Patel, Emily Rivera, Laura Weisbein, Ahmed Ali, Michael D. Kappelman, Brian B. Koo

**Affiliations:** 1 Department Neurology, Yale School of Medicine, New Haven, CT USA; 2 Yale School of Public Health, New Haven, CT USA; 3 Yale College, New Haven, CT USA; 4 Center for Gastrointestinal Biology and Disease, University of North Carolina School of Medicine, Chapel Hill, NC USA; 5 Department of Pediatrics, University of North Carolina School of Medicine, Chapel Hill, NC USA; 6 Connecticut Veterans Affairs Healthcare System, New Haven, CT USA

**Keywords:** Parkinson's disease, Gastrointestinal diseases, Epidemiology, Inflammation

## Abstract

REM sleep behavior disorder (RBD) and inflammatory bowel disease (IBD) are associated with Parkinson’s Disease. Using a cross-sectional design, we assessed RBD prevalence in IBD and controls. In total, 158 controls and 462 IBD patients completed IBD-related history questionnaires, the RBD Single-Question Screen (RBD1Q), and the RBD-Screening Questionnaire. RBD prevalence was compared using chi-squared testing. Logistic regression explored IBD-related factors associated with RBD (RBD1Q), adjusting for age, sex, race, and antidepressant usage. RBD prevalence was higher in IBD (14.1% vs. 5.1%; *P* = 0.002), driven by differences in women; 13.3% IBD and 1.0% controls had RBD. Among men, 16.0% IBD and 11.9% controls had RBD. IBD was associated with >threefold RBD odds (OR = 3.08, 95% CI [1.43, 6.62], *P* = 0.003). Men with RBD and IBD had higher IBD-related hospitalization rates than IBD males without RBD (95.2% vs. 63.0%; *P* = 0.004). RBD is more prevalent in IBD than in controls and is associated with severe IBD.

## Introduction

Parkinson’s disease (PD) is the second most common neurodegenerative disease in the U.S., with a prevalence of ~1.0% in adults over 60 years of age^1^. PD is largely a disease of older adults as α-synuclein deposition in the brain sufficient to cause neurological symptoms occurs over decades^2^. Insight into the pathophysiologic process leading to α-synuclein deposition is needed to identify therapies capable of altering the disease course. PD is best known for its cardinal motor symptoms of bradykinesia, rigidity, and tremor, yet onset of the pathological process may be better marked by non-motor symptoms, such as constipation, hyposmia and dream enactment. Non-motor symptoms usually precede motor manifestations by decades, providing a means by which to identify and intervene in those at high risk for synucleinopathy phenoconversion^3^.

In PD, prodromal symptoms of constipation suggest extraneural pathology in the gut. Indeed, aggregated α-synuclein is expressed in the gut and brain of PD patients^4^. Braak’s hypothesis suggests that synucleinopathy begins in the gut, then travels via the vagus nerve to the lower brainstem eventually reaching the substantia nigra and neocortex to cause PD-related neurological symptoms^5^. In PD patients, fecal microbiome dysbiosis is associated with aggregated α-synuclein deposition and colonic inflammation^6^. Moreover, PD incidence is increased in those with gastrointestinal conditions, particularly inflammatory bowel disease (IBD)^7^. IBD is a gastrointestinal disorder with a global prevalence of 0.3–0.5% in which affected individuals suffer bowel inflammation, causing recurrent severe bouts of abdominal pain, constipation and diarrhea^8^. IBD patients have increased α-synuclein aggregation in the gut^9^. Due to the higher PD prevalence among IBD patients and the shared pathological synuclein aggregation, IBD is postulated to be a risk factor for PD^10^.

Among the constellation of non-motor prodromal PD symptoms, dream enactment is most striking, occurring in parasomnia, REM behavior disorder (RBD), in which individuals act out their dreams, often in a violent manner as the muscle atonia of REM sleep is lost. RBD, which affects 0.5% to 2% of the general population^11^, is the single most powerful predictor of synucleinopathy phenoconversion^12^. Prospective studies demonstrate that 73.5% of individuals with RBD will develop synucleinopathy within 12 years, with PD being the most common^12^.

Given the established interconnections among PD, RBD, and IBD, the current study aims to assess RBD prevalence in IBD patients and age–sex matched controls and to identify which IBD-related factors are associated with RBD. While no prior studies have specifically investigated the direct relationship between IBD and RBD, previous research has demonstrated higher rates of poor sleep quality in individuals with IBD compared to healthy controls^13^. Given the high prevalence of poor sleep quality in individuals with RBD, this suggests a potential association between sleep dysfunction and IBD, as well as a possible link between IBD and RBD. Considering the evidence linking gut pathology and PD and the significant predilection for synucleinopathy among those with RBD, we hypothesize that RBD prevalence among IBD patients will exceed that in a control population. By focusing on the prevalence and contributing factors to RBD in the IBD population, our study seeks to provide insight that could pave the way for early identification strategies for patients at risk of synucleinopathy.

## Results

### Baseline characteristics

Between February 2023 and May 2024, 462 individuals with IBD and 158 controls completed online questionnaires. Table 1 shows demographic and medical characteristics of the groups. IBD and control individuals were comparable for age (57.1 ± 10.9 versus 57.4 ± 11.7; *P* = 0.81) and race (95.2% versus 91.1% Caucasian; *P* = 0.09). Sex distribution between the groups was significantly different in that the IBD group had 71.6% females versus 62.6% among controls (*P* = 0.04). The groups were comparable in regard to comorbidities, including hypertension, diabetes mellitus, coronary artery disease, and depression. IBD individuals showed a higher prevalence of antidepressant usage compared to controls (22.1% versus 12.7%, *P* = 0.01). Only two individuals with IBD and one control screened positive for PD, which was not statistically different.

**Table 1:** Subject demographics, characteristics, and comorbidities

	IBD (*N* = 462)	Controls (*N* = 158)	*P* value
Age (SD)	57.1 (10.9)	57.4 (11.7)	0.81
Sex			0.04
Female (*n*, %)	331 (71.6%)	99 (62.6%)	
Male (*n*, %)	131 (29.4%)	59 (37.4%)	
Race			0.09
White (*n*, %)	440 (95.2%)	144 (91.1%)	
Other (*n*, %)	22 (4.8%)	14 (8.9%)	
Hypertension (*n*, %)	103 (22.3%)	33 (20.9%)	0.72
Diabetes mellitus (*n*, %)	20 (4.3%)	9 (5.7%)	0.48
Coronary artery disease (*n*, %)	6 (1.3%)	3 (1.9%)	0.59
Depression (*n*, %)	81 (17.5%)	19 (12.0%)	0.10
Antidepressant usage	105 (22.7%)	20 (12.7%)	0.01

Data are presented as sample size or n (percentage) for categorical variables and mean (standard deviation) for continuous variables. Characteristics between the groups were compared using chi-square testing for categorical variables and Student’s *t* testing for continuous variables.

### RBD prevalence

The prevalence of RBD using various definitions in the IBD and control groups is shown in Table 2. RBD prevalence was significantly higher in the IBD than in the control group across all RBD definitions. Based on high sensitivity and specificity for RBD, we primarily used the RBD1Q to assess RBD prevalence. Based upon this, RBD prevalence was significantly higher in the IBD than the control group (14.1% vs. 5.1%; *P* = 0.002). Similarly, RBD prevalence was higher in those with IBD compared to age–sex matched controls when RBD-SQ, restrictive RBD, and liberal RBD definitions were used. Upon excluding two IBD participants and one control with a diagnosis of PD, results were largely unchanged with RBD prevalence being significantly higher in the IBD than control group (13.9% vs. 5.1%; *P* = 0.003).

**Table 2:** RBD prevalence in IBD and control groups using various RBD screening scales

	IBD (*n* = 462)	Controls (*n* = 158)	*P* value
RBD1Q (*n*, %)	14.1%	5.1%	0.002
RBD-SQ (*n*, %)	20.0%	11.4%	0.01
RBD liberal (*n*, %)	24.1%	12.7%	0.002
RBD restrictive (*n*, %)	10.0%	3.8%	0.015

*RBD1Q* REM Sleep Behavior Single-Question Screen, *RDB-SQ* REM Sleep Behavior Disorder Screening Questionnaire, *RBD Liberal* a positive score on RBD1Q or RBD-SQ, *RBD Restrictive* a positive score on RBD1Q and RBD-SQ.

Statistical test: Chi-squared.

By further categorizing the IBD group into CD and UC, we observed that RBD prevalence remained higher in both IBD subtypes compared to controls; however, RBD prevalence was similar between the CD and UC groups. A two-sided Fisher’s Exact Test showed a statistically significant association between sex and RBD prevalence across IBD subtype and controls (*P* = 0.004). Chi-squared testing revealed differences of RBD prevalence among control, UC and CD groups (*P* = 0.01), largely driven by very low prevalence of IBD in female controls. Specifically, there is a significant association between RBD status across IBD type and controls (*P* = 0.002) among female participants (Table 3). This significantly higher prevalence of RBD was not observed in male participants with IBD (*P* = 0.477), but, although not statistically significant, RBD seemed to occur more frequently in CD compared to UC in men (18.2% vs. 11.9%; *P* = 0.16).

**Table 3:** RBD prevalence categorized by group and sex

	Total IBD (*n* = 462)	Crohn’s disease (*n* = 298)	Ulcerative Colitis (*n* = 164)	Controls (*n* = 158)	*P* value
Female (*n*, %)	44 (13.3%)	26 (12.4%)	18 (14.9%)	1 (1.0%)	0.002
Male (*n*, %)	21 (16.0%)	16 (18.2%)	5 (11.9%)	7 (11.9%)	0.47
Total (*n*, %)	65 (14.1%)	42 (14.1%)	23 (14.0%)	8 (5.1%)	0.01

This table shows the prevalence of RBD in the following groups: Total IBD, Crohn’s Disease, Ulcerative Colitis, and Controls. The presence of RBD was defined by a “Yes” on the RBD1Q. The data is categorized by sex, displaying the number of females and males with RBD for each condition, along with the prevalence of RBD as a percentage. Statistical test: Chi-squared among Crohn’s disease, ulcerative colitis, and controls. The *P* value in each row reflects the chi-squared test conducted for the corresponding sex in that row.

Logistic regression assessed which factors, including IBD, age, Caucasian race, sex, and antidepressant usage, were associated with RBD in the overall cohort. The outcome variable, RBD, was defined by the RBD1Q. Shown in Table 4, IBD was significantly associated with a more than threefold odds of having RBD (OR = 3.08, 95%, CI [1.43, 6.62]), while age, race, sex, and antidepressant usage were not associated with RBD. Men seem to show higher odds of RBD than women (OR = 1.64, 95% CI [0.97, 2.76]). In a sensitivity analysis, self-report of the comorbidities of coronary artery disease, diabetes and hypertension were added into the logistic regression model, and results were not appreciably different with IBD continuing to be significantly associated with RBD with an OR ~3 (data not shown).

**Table 4:** Logistic regression analysis of factors associated with the presence of RBD

	Odds ratio	95% confidence interval
IBD status: yes	3.08	1.43, 6.62
Age	1.00	0.98, 1.03
Caucasian: yes	1.28	0.37, 4.36
Sex: male	1.64	0.97, 2.76
Antidepressant usage: yes	1.47	0.82, 2.61

Notes: The table displays odds ratios and 95% confidence intervals for variables influencing the odds of the presence of RBD, adjusted for sex, age, race (Caucasian), and antidepressant usage.

### IBD characteristics and RBD

Among the IBD cohort, the frequency of IBD-related characteristics, such as IBD hospitalizations, IBD intestinal surgery, ostomy, and TNF-α inhibitor treatment, are categorized by the presence of RBD (Table 5). The ostomy rate was significantly higher among RBD than non-RBD individuals (*P* = 0.049). No significant differences were observed in the overall number of hospitalizations, intestinal surgeries, TNF-α inhibitor treatment, IBD duration, short CDAI, or SSCI between those with and without RBD.

**Table 5:** Prevalence of IBD-related characteristics between RBD vs non-RBD

	RBD (*n* = 65)	Non-RBD (*n* = 396)	*P* value
IBD hospitalized			
All	73.8%	61.7%	0.059
Female	63.6%	61.2%	0.76
Male	95.2%	63.0%	0.004***
Intestinal surgery			
All	49.2%	47.5%	0.79
Female	38.6%	44.9%	0.43
Male	71.4%	54.1%	0.14
Ostomy			
All	12.5%	5.9%	0.049*
Female	9.1%	4.6%	0.21
Male	20.0%	9.2%	0.15
TNF treatment			
All	58.5%	53.4%	0.45
Female	56.8%	53.0%	0.63
Male	61.9%	54.5%	0.53
Years of IBD			
All	27.6 ± 15.1	26.8 ± 13.3	0.69
Female	24.8 ± 14.5	25.2 ± 12.2	0.85
Male	33.4 ± 15.2	30.8 ± 15.3	0.48

This table compares rates of IBD-related intestinal surgeries and hospitalizations between patients with and without RBD. The table is categorized by sex, displaying the percentage of participants in the IBD group, with and without RBD, who have undergone Intestinal Surgery, have a current Ostomy, have ever had TNF treatment, and Hospitalizations for IBD (IBD Hospitalized). Statistical significance levels are denoted as follows: ***P < 0.001, *P < 0.05.

When further sub-categorized by sex, males with both IBD and RBD have significantly greater rates of IBD-related hospitalization compared to non-RBD males (*P* = 0.004). No significant differences were observed in ostomy rates, intestinal surgery or TNF-α inhibitor treatment among males with and without RBD. However, men with RBD seem to more frequently have had IBD-related surgery (71.4% vs. 54.1%; *P* = 0.14). These findings could suggest that IBD severity is related to the presence of RBD at least in men. Among females, there was no significant difference in the prevalence of IBD-related hospitalizations, intestinal surgeries, ostomy, or TNF-α inhibitor treatment between those with and without RBD.

## Discussion

Although both RBD and IBD are associated with incident synucleinopathy^12,14^, there are no published reports on the occurrence of RBD in IBD patients. Thus, our study is the first to assess whether RBD prevalence is higher in IBD patients than among age–sex-matched controls. This study’s main finding is that RBD prevalence in IBD patients is significantly higher than in healthy controls (14.1% vs. 5.1%; *P* = 0.002). In fact, IBD was associated with threefold higher odds of having RBD, while neither age nor sex was associated with RBD. The difference in RBD prevalence was driven by a large change seen in women, in whom 13.3% of IBD females and only 1.0% of female controls had RBD. Among the IBD cohort, IBD-related hospitalizations were higher in those with RBD, and significantly so in men with RBD compared to men without RBD. While surgeries were not significantly more frequent in IBD patients with RBD, men with IBD and RBD seem to show higher rates of IBD-related surgery than men with IBD without RBD. These findings suggest that IBD severity increases the risk for RBD, at least in men. At the same time, the presence of IBD appears to narrow the male predominance in RBD prevalence, as the male-to-female ratio of those with RBD was much less among IBD sufferers than controls.

RBD is a disease in which patients “act out their dreams,” in that the exhibited behavior mirrors dream content. RBD is the strongest clinical predictor of incident synucleinopathy and is associated with poor prognosis in PD^15^. RBD has more predictive strength and specificity than other prodromal markers (e.g., hyposmia, constipation); 50–90% of RBD patients will eventually develop synulceinopathy^16^.

On the other hand, prodromal PD symptoms like constipation suggest that pathology also arises in the gut. Individuals with gut inflammation, particularly IBD patients, demonstrate higher PD risk^7,10^, and thus may also be at increased risk of RBD. This interconnected relationship among IBD, PD, and RBD may reflect the role of the gut–brain axis in mediating the onset and progression of synucleinopathy. Other mechanisms by which gut-related pathology can travel to the brain are through immune cell (e.g., T or B cell) trafficking from the gut into the systemic circulation and brain^17^. Peripheral inflammation and its associated inflammatory mediators contribute to increased intestinal permeability, facilitating entry of enterotoxins and immune cells into the systemic circulation^18^. Similarly, inflammatory mediators, like interleukin-1β, result in increased blood–brain-barrier permeability through tight junction protein downregulation^19^, putatively increasing immune cell trafficking. Yet another mechanism by which the gut communicates with the brain is through metabolites produced by gut microbiota that enter into the systemic circulation and cross the blood–brain-barrier^20^. This gut–brain crosstalk occurs under normal circumstances, but is pathologically altered in individuals with IBD, exposing the brain to toxic metabolites, predisposing to neurodegeneration^21^.

By showing an association between IBD and RBD, our study suggests that gut–brain crosstalk can result in brain pathology. However, it should be noted that given our study’s cross-sectional design, we cannot establish a causal or serial relationship between RBD and IBD. In addition, our study did not assess potential PD biomarkers (e.g., α-synuclein), making it challenging to implicate specific pathologies driving the progression of IBD to RBD. Nevertheless, it is plausible that IBD could lead to brain synucleinopathy through any of the mechanisms outlined in the preceding paragraph. First, there is ample evidence of synucleinopathy not only in the gut of IBD patients, but also in the mesencephalon, even in the absence of RBD or PD sympatomatology^22^. In addition, CD4+ T lymphocytic cells, prominent in the gut of mice with chronic colitis, infiltrate the blood–brain-barrier and enter the brain^23^. Finally, intestinal microbial dysbiosis, prevalent in IBD, can result in decreased neuroprotective metabolites and increased neurotoxic microbiome metabolites^24^. Indeed, this pattern of reduced beneficial fatty acid production and increased neurotoxin has been shown in PD patients^25^.

To better assess whether IBD confers risk for RBD, we examined IBD-related characteristics, including hospitalizations, disease duration, intestinal surgery, ostomy, and TNF-α inhibitor treatment. When comparing individuals with both IBD and RBD to those with IBD alone, we found no significant differences in hospitalizations, disease duration, intestinal surgery, or TNF-α inhibitor treatment. We did observe a significantly higher prevalence of ostomy in those with both IBD and RBD. Notably, when analyzed by sex, males with both IBD and RBD had a significantly higher rate of IBD hospitalizations than IBD males without RBD and seemed to have more IBD-related surgery. Sex differences in IBD-related factors in our study are consistent with previously described sex variations in IBD severity and presentation. Prior studies report that males are more likely to undergo IBD-related surgery^26^, have a higher risk of hospital readmission, while females are more likely to experience bowel obstruction, pouch-related fistulas, increased bowel frequency, and extraintestinal manifestations^27^. The difference in RBD prevalence between IBD patients and controls was driven by large differences in females. The reasons accounting for this sex difference is unclear, but it may relate to the known predilection for extraintestinal manifestations in women, including ocular manifestations, arthritis, erythema nodosum, and rheumatoid arthritis^28^. RBD may be yet another IBD-related extraintestinal manifestation.

The association we observed between RBD and IBD emphasizes the growing evidence of gastrointestinal inflammation in PD pathophysiology. Elevated levels of pro-inflammatory cytokines, like Interleukin-1β, Interleukin-6, C-reactive protein, and TNF-α, have been found in blood, stool, and CSF of PD patients, paralleling the pro-inflammatory cytokine profile in IBD^29–31^. Calprotectin, a fecal marker of intestinal inflammation, is significantly elevated in PD patients^32^. In addition, both IBD and PD patients have structural alterations in intestinal epithelium triggered by inflammation-prompted glial cell dysfunction^33,34^. The shared pro-inflammatory cytokine signature and gastrointestinal barrier dysfunction observed in individuals with PD and IBD support the premise of gut-to-brain inflammatory progression.

Strengths of the study include its robust methodology. We conducted a cross-sectional study to compare RBD prevalence in controls and IBD patients. Controls were selected from the general population, recruited through emails and flyers, and targeted based upon age, sex, and race as our IBD cohort was predominantly Caucasian, female and 40 years or older. The IBD group was recruited from the IBD Partners registry, a validated patient-powered IBD research network with ~16,000 adult IBD patients. We were careful not to bias responses to RBD-related questions, thus deception was used, making participants unaware of the study’s objective to assess RBD.

Our study does have limitations. First and foremost, we used questionnaire assessment to establish RBD rather than a gold-standard clinical-polysomnographic diagnosis. The cohort was available only virtually; therefore, sleep testing was not feasible. As a result, we utilized the validated RBD1Q, though we acknowledge that this may have led to an overestimation of RBD prevalence in both groups, and we cannot exclude the possibility of differential misclassification. To mitigate this risk, we also employed the RBD-SQ and examined RBD prevalence using restrictive and liberal RBD definitions. Furthermore, we acknowledge that our cohort of IBD patients and controls is ultimately a convenience sample, and the actual prevalence of RBD may not fully represent the overall IBD population and/or the general U.S. population. Challenges in recruiting an age–sex matched control group resulted in a smaller number of controls than cases. In addition, limited racial diversity of the groups (Caucasian predominance) decreases the generalizability of our findings. While there was a slight sex distribution disparity with controls being disproportionately more male, this difference would be expected to result in RBD being more common in controls, as men are more likely to have RBD.

RBD is a complex and multifaceted sleep disorder influenced by several confounding factors that can affect its detection and diagnosis, including sleeping alone, the presence of other sleep disorders, and the use of antidepressants. Individuals who live alone may be less likely to recognize or report symptoms, as there is no bed partner to observe abnormal dream-enactment behaviors. Our study did not collect data on whether participants lived alone or with a spouse, which we acknowledge as a limitation and a potential area for future research. In addition, coexisting sleep disorders, such as obstructive sleep apnea or insomnia, can obscure or mimic RBD symptoms, further complicating an accurate diagnosis. Self-report sleep apnea and insomnia rates were low (less than five in both groups), were not significantly different between the groups and did not appreciably affect the association between IBD status and RBD. The use of antidepressants, particularly selective serotonin reuptake inhibitors, has also been linked to the onset of RBD-like behaviors. While significantly more IBD patients were taking an antidepressant than controls, antidepressant usage was not associated with RBD and did not appreciably affect the association between IBD status and RBD.

Our study’s finding of higher RBD prevalence among individuals with IBD extends the understanding of how gastrointestinal inflammation may contribute to neurodegeneration, aligning with the broader PD literature. Notably, the shared pro-inflammatory cytokine profiles and epithelial barrier dysfunction observed in IBD and PD suggest a possible mechanistic link, supporting hypotheses about the role of gut–brain interactions in neurodegeneration. The results of this study underscore the need for further investigation of connections among gut health, sleep, and neurological conditions, which could ultimately inform strategies for intervention in affected populations. Future research may focus on longitudinal studies that track IBD progression, sleep disturbance, and neurodegenerative markers longitudinally. In addition, studying the gut microbiome in patients with concomitant RBD and IBD could provide insight into which bacterial species may underlie pathological gut–brain crosstalk, leading to gut and brain synucleinopathy.

## Methods

### Study population and recruitment

We conducted a cross-sectional study in individuals with IBD and age–sex matched controls to determine RBD prevalence and IBD-related factors predicting RBD. From February through November of 2023, we enrolled 462 English-speaking adults with IBD from the IBD Partners Patient Powered Research Network, a Crohn’s & Colitis Foundation registry of approximately 16,000 IBD patients, who have reported details on their health, healthcare, and outcomes using internet-based surveys. Further cohort details have been published previously^35^. IBD Partners registrants have self-reported their IBD diagnosis. A prior IBD Partners cohort validation study demonstrated highly accurate IBD self-report diagnosis, as 97% of participants had a physician-confirmed IBD diagnosis^36^.

To provide a basis for comparison, 158 English-speaking control participants without IBD were recruited from July 2023 through May 2024 through email and research flyer solicitation. Research flyers were distributed in the community and handed out in person. Flyers explaining the study contained a QR code linked to the online questionnaire. When handing out flyers, Caucasian persons older than 40 years were targeted to closely match our IBD cohort demographics. In order to obtain unbiased responses to dream-enactment questions, deception was used in that communication stated that the study’s purpose was to evaluate whether certain lifestyle factors, like exercise, work, and sleep, affect quality of life in persons with IBD compared to controls. Participants were unaware that the study’s main aim was to determine the prevalence and correlates of RBD.

Study-specific eligibility criteria for both IBD and control participants included age ≥40 years, English literacy, and internet access. Exclusion criteria included history of post-traumatic stress disorder or narcolepsy as dream enactment occurs in both entities. All participants completed online surveys on REDCap, collecting data on demographics, dream enactment, sleep quality, PD-related symptoms, IBD history, and medical history.

### Standard protocol approvals and patient consents

Before beginning the survey, a written description of the study and its risks was provided for potential participants to read. The survey was anonymous for control participants, who acknowledged that they understood their involvement and electronically agreed to participate, thereby providing informed consent. IBD participants provided written informed consent electronically. The study received necessary approvals from the Yale Institutional Review Board.

### Assessment of IBD

The presence of IBD was identified by a single question on the IBD Questionnaire: “Have you been diagnosed with Crohn’s disease (CD), Ulcerative colitis (UC) or indeterminate colitis/IBD unclassified by a physician specializing in gastrointestinal disorders?”, in which “Yes” indicated prevalent IBD. For individuals with IBD, data were gathered on diagnosis year, IBD symptom severity, IBD-related medications, and the presence IBD-related surgeries or hospitalizations.

IBD participants also specified IBD subtype, CD, UC, or Indeterminate colitis/IBD unclassified. CD patients completed the Short CD Activity Index (Short CDAI), which evaluates frequency of liquid or soft stools daily and ratings of abdominal pain and general well-being; scores range from 0 to 450 with higher scores indicating greater disease severity^37^. UC or indeterminate colitis patients completed the Simple Clinical Colitis Activity Index (SCCI), which contains five items assessing daytime/nocturnal bowel frequency, rectal bleeding, defecation urgency, and general well-being; scores range from 0 to 19 with greater scores indicating more severe disease^38^.

### Assessment of RBD

Both IBD participants and controls completed questionnaires assessing the presence of RBD through two separate instruments. The REM Sleep Behavior Disorder Single-Question Screen (RBD1Q) uses one question to assess RBD. This question, “Have you ever been told, or suspected yourself, that you seem to ‘act out your dreams’ while asleep (for example, punching, flailing your arms in the air, making running movements, etc.)?”, if responded to as “Yes” constituted RBD. RBD1Q is validated and has 93.8% sensitivity and 87.2% specificity compared to the gold-standard clinical polysomnographic diagnosis^39^. We also used the RBD Screening Questionnaire (RBD-SQ), which consists of ten questions, pertaining to dreams and dream enactment, with scoring based on yes/no responses^40^. Summing “Yes” responses yields a total RBD-SQ score if ≥5 indicates RBD. RBD-SQ has a sensitivity of 80% and specificity of 55%^41^. The presence of RBD was mainly defined by RBD1Q, based upon its high sensitivity and specificity^39^. We also employed a liberal RBD definition for positive scores on either RBD1Q or RBD-SQ; and a restrictive RBD definition for positive scores on both RBD1Q and RBD-SQ with the former used to optimize sensitivity and the latter to optimize specificity. To address potential confounding factors affecting the assessment of RBD, such as age, sex, race, antidepressant usage, and coexisting sleep disorders, we collected participant-reported data on these factors as a part of their General Medical Questionnaire (see “Other questionnaires” section).

### Other questionnaires

Both IBD and controls completed the following questionnaires. The General Medical Questionnaire gathers demographic details, including age, sex, and race, alongside medical history and current medications. The PD Screening Scale contains 8 self-reported items, screening for PD. Questions included: (1) “Do you have tremors when sitting quietly?”; (2) “Do you notice that you do things stiffly or slowly?”; (3) “Do you have a shuffling gait?”; (4) “Do you have a stooped posture?”; (5) “Do you have decreased arm swing when walking?”; (6/7) “Were you seen by a physician/neurologist for this condition and if so was the diagnosis Parkinson disease?” PD was considered when participants answered “Yes” to (1) and (2) and one of (3), (4) or (5), and one of (6) or (7). This scale was previously used to assess the accuracy of PD family history in a PD family cohort study^42^.

### Statistical analysis

Baseline descriptive characteristics were summarized for the total study population and compared across predefined strata, including sex and group. Categorical variables were compared using Fisher’s Exact Test when cell counts were less than five, and Pearson’s Chi-squared test otherwise. Continuous variable means were compared using Student’s *t* testing. Using a pooled probable RBD prevalence of 5.65% from a published meta-analysis^11^ and estimate of RBD prevalence in IBD of 15.0%, to achieve 80% power with a 0.05 two-tailed alpha, sample sizes of 165 in each group would be required. Recruitment was carried out with this power calculation in mind.

Prevalence of RBD in IBD patients and controls was assessed using the RBD1Q and RBD-SQ. Liberal and strict RBD definitions were positive on either scale and positive on both scales, respectively. For each RBD definition, RBD prevalence was summarized as the percentage of persons with RBD over the total number of individuals in each group (IBD or control). Differences in RBD prevalence between IBD and control groups were assessed using Pearson’s Chi-squared testing. Fisher’s Exact Test assessed whether or not RBD was differentially expressed among IBD subsets (Crohn’s and UC) and controls by sex. Chi-squared tests were then used to determine in what sex and group, there was differential RBD expression. Given that PD is associated with both IBD and RBD, we performed a sensitivity analysis excluding those with a diagnosis of PD (2 in the IBD group and 1 in the control group) assessing the prevalence of RBD in IBD and control groups using Pearson’s Chi-squared testing.

Missing data was omitted from the statistical analysis. Standards of Reporting of Neurological Disorders (STROBE) was used to guide this study and its reporting^43^.

To further assess the relationship between IBD (main variable) and the outcome, RBD presence, logistic regression models were constructed with covariates of sex, age, being Caucasian, and antidepressant usage. Subsequent analysis was conducted among the IBD cohort only to assess IBD-related factors influencing RBD prevalence. First, IBD-related characteristics (hospitalizations, intestinal surgeries, ostomy, and TNF-α inhibitor treatment) between individuals with and without RBD were compared using Pearson’s Chi-square or Fisher’s Exact test. Logistic regression then examined which IBD characteristics (IBD type, IBD surgery, number of hospitalizations) are associated with odds of having RBD, while also controlling for age, sex, and Caucasian race. Interaction between sex and number of hospitalizations was included to evaluate whether the impact of hospitalization frequency on RBD prevalence varied by sex. Odds ratios (ORs) with 95% confidence intervals (CIs) were calculated to quantify the strength of associations. Statistical significance was determined with a two-tailed *P* value threshold of 0.05. All analyses were conducted using R software version 4.4.1 (Auckland, New Zealand).

## Supplementary information

Supplemental Materials

## Data Availability

Anonymized data not published within this article will be made available by reasonable request from qualified investigators.

## References

[1] Tysnes, O. B. & Storstein, A. Epidemiology of Parkinson’s disease. *J. Neural Transm.* **124**, 901–905 (2017). 28150045 10.1007/s00702-017-1686-y

[2] Beauchamp, L. C. et al. Using (18)F-AV-133 VMAT2 PET imaging to monitor progressive nigrostriatal degeneration in Parkinson disease. *Neurology* **101**, e2314–e2324 (2023). 37816639 10.1212/WNL.0000000000207748 PMC10727223

[3] Fereshtehnejad, S. M. et al. Evolution of prodromal Parkinson’s disease and dementia with Lewy bodies: a prospective study. *Brain* **142**, 2051–2067 (2019). 31111143 10.1093/brain/awz111

[4] Lee, H. S., Lobbestael, E., Vermeire, S., Sabino, J. & Cleynen, I. Inflammatory bowel disease and Parkinson’s disease: common pathophysiological links. *Gut* **70**, 408–417 (2021). 33067333 10.1136/gutjnl-2020-322429

[5] Braak, H. et al. Staging of brain pathology related to sporadic Parkinson’s disease. *Neurobiol. Aging* **24**, 197–211 (2003). 12498954 10.1016/s0197-4580(02)00065-9

[6] Keshavarzian, A. et al. Colonic bacterial composition in Parkinson’s disease. *Mov. Disord.* **30**, 1351–1360 (2015). 26179554 10.1002/mds.26307

[7] Zhu, F. et al. The risk of Parkinson’s disease in inflammatory bowel disease: a systematic review and meta-analysis. *Dig. Liver Dis.* **51**, 38–42 (2019). 30309751 10.1016/j.dld.2018.09.017

[8] Ng, S. C. et al. Worldwide incidence and prevalence of inflammatory bowel disease in the 21st century: a systematic review of population-based studies. *Lancet* **390**, 2769–2778 (2017). 29050646 10.1016/S0140-6736(17)32448-0

[9] Prigent, A. et al. Enteric alpha-synuclein expression is increased in Crohn’s disease. *Acta Neuropathol.* **137**, 359–361 (2019). 30506319 10.1007/s00401-018-1943-7

[10] Villumsen, M., Aznar, S., Pakkenberg, B., Jess, T. & Brudek, T. Inflammatory bowel disease increases the risk of Parkinson’s disease: a Danish nationwide cohort study 1977-2014. *Gut* **68**, 18–24 (2019). 29785965 10.1136/gutjnl-2017-315666

[11] Cicero, C. E. et al. Prevalence of idiopathic REM behavior disorder: a systematic review and meta-analysis. *Sleep* **44**, zsaa294 (2021). 10.1093/sleep/zsaa294 33388771

[12] Postuma, R. B. et al. Risk and predictors of dementia and Parkinsonism in idiopathic REM sleep behaviour disorder: a multicentre study. *Brain* **142**, 744–759 (2019). 30789229 10.1093/brain/awz030 PMC6391615

[13] Zhang, Y. et al. Sleep characteristics and influencing factors of sleep quality in patients with inflammatory bowel disease-peripheral arthritis. *Front. Med.* **6**, 190 (2019). 10.3389/fmed.2019.00190 PMC6956445 31970156

[14] Fu, P., Gao, M. & Yung, K. K. L. Association of intestinal disorders with Parkinson’s disease and Alzheimer’s disease: a systematic review and meta-analysis. *ACS Chem. Neurosci.* **11**, 395–405 (2020). 31876406 10.1021/acschemneuro.9b00607

[15] Shrestha, N. et al. The correlation between Parkinson’s disease and rapid eye movement sleep behavior disorder: a systematic review. *Cureus* **13**, e17026 (2021). 34522507 10.7759/cureus.17026 PMC8425494

[16] Jin, H., Zhang, J. R., Shen, Y. & Liu, C. F. Clinical significance of REM sleep behavior disorders and other non-motor symptoms of Parkinsonism. *Neurosci. Bull.* **33**, 576–584 (2017). 28770440 10.1007/s12264-017-0164-8 PMC5636735

[17] Cabezudo, D., Tsafaras, G., Van Acker, E., Van den Haute, C. & Baekelandt, V. Mutant LRRK2 exacerbates immune response and neurodegeneration in a chronic model of experimental colitis. *Acta Neuropathol.* **146**, 245–261 (2023). 37289222 10.1007/s00401-023-02595-9 PMC10328902

[18] Craig, C. F. et al. Neuroinflammation as an etiological trigger for depression comorbid with inflammatory bowel disease. *J. Neuroinflammation.* **19**, 4 (2022). 34983592 10.1186/s12974-021-02354-1 PMC8729103

[19] Wang, Y. et al. Interleukin-1β induces blood-brain barrier disruption by downregulating Sonic hedgehog in astrocytes. *PLoS ONE* **9**, e110024 (2014). 25313834 10.1371/journal.pone.0110024 PMC4196962

[20] Ahmed, H. et al. Microbiota-derived metabolites as drivers of gut-brain communication. *Gut Microbes* **14**, 2102878 (2022). 35903003 10.1080/19490976.2022.2102878 PMC9341364

[21] Heinzel, S. et al. Elevated fecal calprotectin is associated with gut microbial dysbiosis, altered serum markers and clinical outcomes in older individuals. *Sci. Rep.* **14**, 13513 (2024). 38866914 10.1038/s41598-024-63893-0 PMC11169261

[22] Espinosa-Oliva, A. M. et al. Inflammatory bowel disease induces pathological α-synuclein aggregation in the human gut and brain. *Neuropathol. Appl. Neurobiol.* **50**, e12962 (2024). 38343067 10.1111/nan.12962

[23] Mickael, M. E. et al. RORγt-expressing pathogenic CD4(+) T cells cause brain inflammation during chronic colitis. *J. Immunol.* **208**, 2054–2066 (2022). 35379749 10.4049/jimmunol.2100869 PMC10103644

[24] Banfi, D. et al. Impact of microbial metabolites on microbiota-gut-brain axis in inflammatory bowel disease. *Int. J. Mol. Sci.* **22**, 1623 (2021). 10.3390/ijms22041623 PMC7915037 33562721

[25] Shao, Y. et al. Comprehensive metabolic profiling of Parkinson’s disease by liquid chromatography-mass spectrometry. *Mol. Neurodegener.* **16**, 4 (2021). 33485385 10.1186/s13024-021-00425-8 PMC7825156

[26] Rustgi, S. D., Kayal, M. & Shah, S. C. Sex-based differences in inflammatory bowel diseases: a review. *Ther. Adv. Gastroenterol.* **13**, 1756284820915043 (2020). 10.1177/1756284820915043 PMC7236567 32523620

[27] Khrom, M. et al. Sex-dimorphic analyses identify novel and sex-specific genetic associations in inflammatory bowel disease. *Inflamm. Bowel Dis.* **29**, 1622–1632 (2023). 37262302 10.1093/ibd/izad089 PMC10547236

[28] Xu, L., Huang, G., Cong, Y., Yu, Y. & Li, Y. Sex-related differences in inflammatory bowel diseases: the potential role of sex hormones. *Inflamm. Bowel Dis.* **28**, 1766–1775 (2022). 35486387 10.1093/ibd/izac094

[29] Houser, M. C. et al. Stool immune profiles evince gastrointestinal inflammation in Parkinson’s disease. *Mov. Disord.* **33**, 793–804 (2018). 29572994 10.1002/mds.27326 PMC5992021

[30] Chen, H., O’Reilly, E. J., Schwarzschild, M. A. & Ascherio, A. Peripheral inflammatory biomarkers and risk of Parkinson’s disease. *Am. J. Epidemiol.* **167**, 90–95 (2008). 17890755 10.1093/aje/kwm260

[31] Devos, D. et al. Colonic inflammation in Parkinson’s disease. *Neurobiol. Dis.* **50**, 42–48 (2013). 23017648 10.1016/j.nbd.2012.09.007

[32] Schwiertz, A. et al. Fecal markers of intestinal inflammation and intestinal permeability are elevated in Parkinson’s disease. *Parkinsonism Relat. Disord.* **50**, 104–107 (2018). 29454662 10.1016/j.parkreldis.2018.02.022

[33] Clairembault, T. et al. Structural alterations of the intestinal epithelial barrier in Parkinson’s disease. *Acta Neuropathol. Commun.* **3**, 12 (2015). 25775153 10.1186/s40478-015-0196-0 PMC4353469

[34] Clairembault, T., Leclair-Visonneau, L., Neunlist, M. & Derkinderen, P. Enteric glial cells: new players in Parkinson’s disease?. *Mov. Disord.* **30**, 494–498 (2015). 25100667 10.1002/mds.25979

[35] Long, M. M. D. et al. Development of an internet-based cohort of patients with inflammatory bowel diseases (CCFA partners): methodology and initial results. *Inflamm. Bowel Dis.* **18**, 2099–2106 (2012). 22287300 10.1002/ibd.22895

[36] Randell, R. L. et al. Validation of an internet-based cohort of inflammatory bowel disease (CCFA partners). *Inflamm. Bowel Dis.* **20**, 541–544 (2014). 24451221 10.1097/01.MIB.0000441348.32570.34 PMC4112538

[37] Thia, K. et al. Short CDAI: development and validation of a shortened and simplified Crohn’s disease activity index. *Inflamm. Bowel Dis.* **17**, 105–111 (2011). 20629100 10.1002/ibd.21400

[38] Higgins, P. D. et al. The quantitative validation of non-endoscopic disease activity indices in ulcerative colitis. *Aliment Pharm. Ther.* **25**, 333–342 (2007). 10.1111/j.1365-2036.2006.03205.x 17269991

[39] Postuma, R. B. et al. A single-question screen for rapid eye movement sleep behavior disorder: a multicenter validation study. *Mov. Disord.* **27**, 913–916 (2012). 22729987 10.1002/mds.25037 PMC4043389

[40] Stiasny-Kolster, K. et al. The REM sleep behavior disorder screening questionnaire—a new diagnostic instrument. *Mov. Disord.* **22**, 2386–2393 (2007). 17894337 10.1002/mds.21740

[41] Bolitho, S. J. et al. Investigating rapid eye movement sleep without atonia in Parkinson’s disease using the rapid eye movement sleep behavior disorder screening questionnaire. *Mov. Disord.* **29**, 736–742 (2014). 24619826 10.1002/mds.25832

[42] Marder, K. et al. Accuracy of family history data on Parkinson’s disease. *Neurology* **61**, 18–23 (2003). 12847150 10.1212/01.wnl.0000074784.35961.c0

[43] von Elm, E. et al. The Strengthening the Reporting of Observational Studies in Epidemiology (STROBE) statement: guidelines for reporting observational studies. *PLoS Med.* **4**, e296 (2007). 17941714 10.1371/journal.pmed.0040296 PMC2020495

